# Validation of Nomograms for Survival and Metastases after Hysterectomy and Adjuvant Therapy in Uterine Cervical Cancer with Risk Factors

**DOI:** 10.1155/2017/2917925

**Published:** 2017-04-27

**Authors:** Won Sup Yoon, Dae Sik Yang, Jung Ae Lee, Nam Kwon Lee, Young Je Park, Chul Yong Kim, Nak Woo Lee, Jin Hwa Hong, Jae Kwan Lee, Jae Yun Song

**Affiliations:** ^1^Department of Radiation Oncology, Ansan Hospital, Korea University College of Medicine, Ansan, Gyeonggido, Republic of Korea; ^2^Department of Radiation Oncology, Guro Hospital, Korea University College of Medicine, Seoul, Republic of Korea; ^3^Department of Radiation Oncology, Anam Hospital, Korea University College of Medicine, Seoul, Republic of Korea; ^4^Department of Obstetrics & Gynecology, Ansan Hospital, Korea University College of Medicine, Ansan, Gyeonggido, Republic of Korea; ^5^Department of Obstetrics & Gynecology, Guro Hospital, Korea University College of Medicine, Seoul, Republic of Korea; ^6^Department of Obstetrics & Gynecology, Anam Hospital, Korea University College of Medicine, Seoul, Republic of Korea

## Abstract

*Background*. Three nomogram models for early stage uterine cervical cancer have been developed (KROG 13-03 for overall survival [OS], SNUH/AMC for disease-free survival [DFS], and KROG 12-08 for distant metastases-free survival [DMFS]) after radical hysterectomy (RH) and pelvic lymph node dissection (PLND). This study aimed to validate these models using our cohort with adjuvant radiotherapy.* Methods*. According to the eligibility criteria of nomogram studies, patients were enrolled in Group A (*N* = 109) for the two KROG models (RH with PLND and whole pelvic irradiation) and Group B (*N* = 101) for the SNUH/AMC model (RH with PLND and squamous histology). Using Cox-regression hazard models, the prognostic factors of our cohorts were evaluated. The risk probabilities induced from published nomogram scores were calculated and the concordance indices were evaluated.* Results*. Group A had 88.1% 5-year OS and 86.0% 5-year DMFS. Group B had 83.0% 5-year DFS. In multivariate analyses, large tumor size for OS (HR 8.62, *P* < 0.001) and DMFS (HR 5.13, *P* = 0.003), young age (≤40 versus 41–64 years) for OS (HR 4.63, *P* = 0.097) and DFS (HR 3.44, *P* = 0.051), and multiple lymph node metastases (0 versus ≥3) for DMFS (HR 4.03, *P* = 0.031) and DFS (HR 3.90, *P* = 0.038) were significantly correlated. The concordance indices for OS, DMFS, and DFS were 0.612 (*P* = 0.002), 0.597 (*P* = 0.014), and 0.587 (*P* = 0.020), respectively.* Conclusion*. The developed nomogram models after RH and PLND are clinically useful in predicting various types of survival with significance.

## 1. Introduction

The incidence of uterine cervical cancer, which was ranked the 4th most common cancer in 1999, dropped to the 7th most common cancer in 2012 in Korean women [1, 2] through multidisciplinary approaches to manage the human papilloma viruses 16 and 18. However, globally, uterine cervical cancer was still the 2nd most common cancer in women in 2012, and uterine cervical cancer remains an important health problem in developing countries [3]. Therefore, persistent efforts to improve the clinical outcome will be continued.

For early uterine cervical cancer of International Federation of Gynecology and Obstetrics (FIGO) stages I-IIA, both surgical resection and radiotherapy are considered as primary therapy. Some gynecologists prefer initial radical hysterectomy (RH), because the ovary function can be preserved in premenopausal women, the gross tumor mass can be removed at once, and treatment time is relatively short. However, there are the risks of recurrence because of remaining micrometastases after RHy, and adjuvant radiotherapy or concurrent chemoradiotherapy (CCRT) are needed to improve survival. In a randomized phase III study, adjuvant radiotherapy reduced the recurrence and progression or death in cases with negative lymph node metastases with deep stromal invasion, lymphovascular invasion, or large tumor diameters (>4 cm) [4, 5]. In addition, adjuvant CCRT improves the survival of patients with any kind of high-risk factors such as positive lymph node metastases, parametrial invasion, and positive margin [6].

The clinical outcomes after standard therapy are associated with various risk factors. The statistical methods, so-called nomograms, translated the various risk factors into a scale and suggest the event or event-free probability for specific disease circumstances according to the summed scales. Using this information, the physician can instantly check the personal prognosis of any patient using a few known factors, predict the clinical outcomes after any therapy, and decide the intensities of subsequent therapies and the necessary frequency of follow-up examinations. While five nomograms has been developed for overall survival (OS) of definite radiotherapy alone or definite CCRT in uterine cervical cancer [7–11], three nomograms including the SNU/AMC for disease-free survival (DFS) [12], KROG 12-08 for distant metastases-free survival (DMFS) [13], and KROG 13-03 for OS [14] have been suggested in the adjuvant therapy setting after RH using various factors such as number of positive lymph nodes (all), parametrial invasion (all), cervical invasion depth (DFS and DMFS), FIGO stage (DFS), age (OS), lymphovascular invasion (OS), combined chemotherapy (OS), and histology (DMFS). Each of the parametrial invasion, histologic type, and FIGO stage except multiple lymph node metastases was the most powerful factor in OS, DMFS, and DFS, respectively. Although each nomogram was based on a huge cohort and the external validation or boot-strap method was followed by internal model construction, further validation using another cohort with a little different treatment circumstances could progress the reliability and the improvement of previous nomogram models by modifying some risk factors.

The validation of nomograms was performed using data of the authors' institutions in uterine cervical cancer after RH and adjuvant radiotherapy

## 2. Methods

### 2.1. Patients

From November 2002 to December 2012, 183 patients received adjuvant radiotherapy after type II or type III RH due to uterine cervical cancer in three branch hospitals of Korea University Medical Center except four patients in one hospital who were enrolled in the KROG study. Our management principle about adjuvant radiotherapy has generally accorded with the practice guideline for gynecology cancer version 2.0 that is recommended by the Korean Society of Gynecologic Oncology [15]. If one or more risk factors such as large tumor size, deep cervical invasion, lymphovascular invasion, positive resection margin, and lymph node metastases were identified, adjuvant radiotherapy was considered. After our Institutional Review Board examined the methodology and ethical adequacy and approved this study (IRB number AS15112), medical records were reviewed.

### 2.2. Distribution of Patients

According to the eligibility criteria of published nomogram studies, our patients were selected and distributed to our Group A and/or Group B. Although there are some minor differences, two KROG studies had similar eligibility criteria and Group A of our study followed their criteria. The eligibility criteria for Group A were FIGO stage I-IIa with adjuvant whole pelvic radiotherapy after RH and pelvic lymph node dissection (PLND). Patients with neoadjuvant chemotherapy before surgery, para-aortic lymph node metastases, or prophylactic irradiation to the para-aortic lymph node, brachytherapy alone, incomplete radiotherapy of below 10 Gy, and small cell or clear cell histology were excluded. Group B was designed to evaluate the SNU/AMC study; therefore our Group B followed the SNU/AMC criteria. The eligibility criteria of Group B were FIGO stages I-IIa with squamous cell carcinoma performing RH and PLND as primary therapy. Patients with neoadjuvant chemotherapy were excluded.

### 2.3. Parameters

The parameters were analyzed in our study including FIGO stage (Ia/Ib/IIa), age group (≤40/41–64/≥65 years), parametrial invasion (negative/positive), invasion thickness into the uterine cervix (≤half/>half), tumor size (≤4/>4 cm), lymphovascular invasion (negative/positive), surgical resection margin (negative/positive), number of lymph node metastases (0/1-2/≥3), histologic type (squamous/nonsquamous), and serum antigen level of squamous cell carcinoma (≤1.5/>1.5 ng/mL). Two of Group A and four of Group B were missing the examination of serum antigen level of squamous cell carcinoma before surgery and were handled as the complete-case method.

### 2.4. Statistics

Survival was calculated from the day of surgery to the event or last follow-up day. The DMFS, DFS, and OS were evaluated by the Kaplan-Meier method as a function of time from the day of surgery. For DMFS and DFS, patients who did not develop distant metastases or any recurrence were censored at the time of their death or at the last available follow-up day, respectively. For OS, patients who were alive at the last available follow-up day were censored. The log-rank tests were used for the comparison of survival differences in terms of clinical factors within the subgroups. The prognostic factors with a *P* value less than 0.2 in univariate analyses, FIGO stage, and the significant prognostic factors in previous nomogram models entered the Cox-regression multivariate analyses using the reword removal method in Group A and Group B. From the multivariate analyses, our hazard function was calculated and used as our model scores. The nomogram scores of DMFS, DFS, and OS were calculated from each case according to the suggestions of the published nomogram models. For OS, the factors of age, lymphovascular invasion, parametrial invasion, the number of lymph node metastases, and concurrent chemotherapy were used. For DMFS, the factors of lymphovascular invasion, histology, parametrial invasion, invasion thickness into the uterine cervix, and the number of lymph node metastases were used. For DFS, FIGO stage, the number of lymph node metastases, parametrial invasion, and invasion thickness into the uterine cervix were used.

The nomograms scores and our Cox-regression models scores were evaluated by the Harrel Concordance index (C-index). A C-index near 0.5 means that the prognostic score is no better than a coin-flip in predicting outcomes. Our study interprets that the values of C-index over 0.8, 0.7, and 0.5 are strong, good, and fair models, respectively. In addition, SPSS 20.0 (IBM SPSS Inc., Chicago, IL) was used to evaluate the log-rank tests and the Cox-regression multivariate analyses. The R software version 3.3.2 was used for the calculation of the C-index. Using packages “survcomp” and “compareC” of the R software version 3.3.2, each C-index was calculated and the C-indies of nomograms scores and our Cox-regression models for each survival were compared, respectively.

## 3. Results

**Figure 1:**
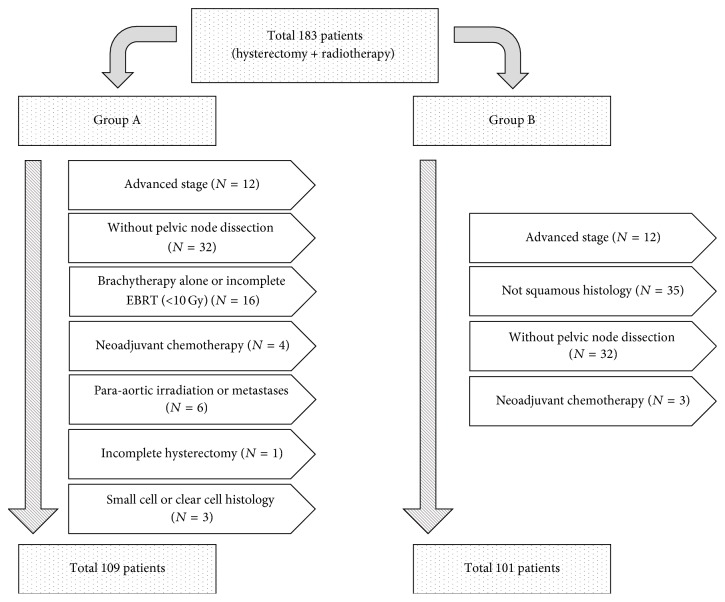
Flowchart of patient selection. Group A for overall survival and distant metastases-free survival and Group B for disease-free survival were developed based on the eligibility criteria for two KROG studies and SNU/AMC study, respectively.

Among a total of 183 patients, 109 patients comprise Group A after excluding advanced stage (*N* = 12), incomplete PLND (*N* = 32), brachytherapy alone or insufficient radiation dose (*N* = 16), neoadjuvant chemotherapy (*N* = 4), para-aortic irradiation or metastases (*N* = 6), incomplete hysterectomy (*N* = 1), and small cell or clear cell histology (*N* = 3). Group B was composed of 101 patients except advanced stage (*N* = 12), nonsquamous histology (*N* = 35), incomplete PLND (*N* = 32), and neoadjuvant chemotherapy (*N* = 3) (Figure 1).

**Figure 2:**
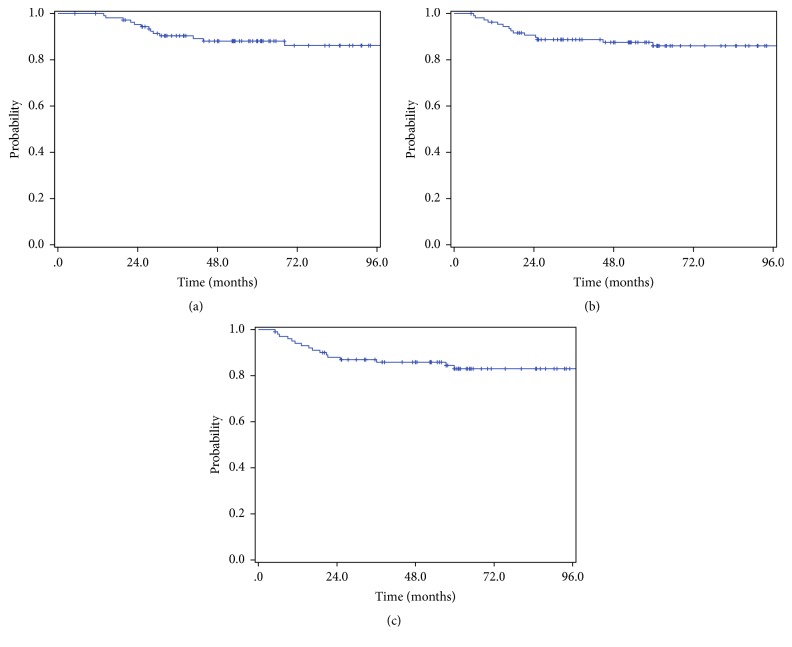
Kaplan-Meier estimates for (a) overall survival (5-year 88.1%), (b) distant metastases-free survival (5-year 86.0%), and (c) disease-free survival (5-year 83.0%).

Patient characteristics of Group A and Group B are presented in Table 1. A median of 50.4 Gy with 28 fractions was given with four box fields as whole pelvic irradiation, and a median of 21 Gy with six fractions was given as brachytherapy. CCRT was done in 73.4% of group A and 69.3% of Group B. The most common regimen was the combination of taxol and cisplatin or carboplatin and the next common regimen was weekly cisplatin. Median follow-up durations were 61.2 and 63.9 months for Group A and Group B, respectively. In Group A, 15 patients died and 14 patients progressed with distant metastases. In Group B, 17 patients experienced disease progression. Therefore, 5-year OS and DMFS were 88.1% and 86.0%, respectively, in Group A, and 5-year DFS was 83.0% in Group B (Figure 2). All of the events of distant metastases developed within 5 years of surgery, and four and one events of OS and DFS, respectively, occurred later than 5 years.

In the log-rank analyses for OS and DMFS, large tumor size (*P* < 0.001, both) and multiple lymph node metastases (0 versus ≥3, *P* = 0.001 and *P* = 0.009, resp.) were significant prognostic factors. Young age (*P* = 0.006, ≤40 versus 41–64) was another significant prognostic factor for OS. Parametrial invasion (*P* = 0.093) and lymphovascular invasion (*P* = 0.080) were marginally related to poor prognosis for OS and DMFS, respectively. Multiple lymph node metastases (*P* = 0.006, 0 versus ≥3) were significantly associated with DFS, and large tumor size (*P* = 0.058), young age (*P* = 0.074, ≤40 versus 41–64), and high serum SCC (*P* = 0.093) were borderline significant (Table 2).

In multivariate analyses, tumor size > 4 cm (HR 8.62 [95% CI 2.73–27.03, *P* = 0.001]) and younger age (HR 4.63 [95% CI 0.83–24.97, *P* = 0.097]) were significant for OS; tumor size > 4 cm (HR 5.13 [95% CI 1.72–15.15, *P* = 0.003]) and multiple lymph node metastases ≥ 3 (HR 4.03 [95% CI 1.13–14.29, *P* = 0.031]) were significant for DMFS; and multiple lymph node metastases ≥ 3 (HR 3.90 [95% CI 1.08–14.07, *P* = 0.038]) and younger age (HR 3.44 [95% CI 0.99–11.91, *P* = 0.051]) were significant for DFS (Table 3).

According to our hazard function from multivariate analyses, the mean function of OS was 0.14 (95% CI 0.11–0.18), that of DMFS was 0.13 (95% CI 0.01–0.16), and that for DFS was 0.17 (95% CI 0.14–0.21). According to each nomogram, the scores of our groups were calculated. The mean value for OS, DMFS, and DFS was 7.6 points (95% CI 6.8–8.4), 57.3 points (95% CI 49.7–65.0), and 99.0 points (95% CI 93.4–104.6), respectively. In the C-index, while our hazard model was 0.671, 0.609, and 0.565 for OS, DMFS, and DFS, respectively, the nomogram scores of our cohort showed a fair correlation, with 0.612 for OS, 0.597 for DMFS, and 0.587 for DFS. Therefore, the nomogram models fairly predicted OS, DMFS, and DFS in our cohort. There were insignificant difference of C-indices between our hazard model and the nomogram scores (Table 4).

## 4. Discussions

Our study reviewed the published nomograms using our cohort. All of the patients of our cohort had undergone RH and radiotherapy or CCRT in three of our branch hospitals; therefore specific techniques of treatments and the indications for adjuvant therapy were relatively uniform. As another merit of our study, each parameter was available without loss in most of the cases. One of our branch hospitals entered the KROG study, but the number of enrolled cases was small, and these cases were excluded from our studies. All of three survival nomogram models predicted well the OS, DMFS, and DFS in our cohort, showing a C-index over 0.58. In addition, there were no statistical difference in our C-index from hazard functions. However, the intensity of predictions decreased to a certain degree in comparison with the original models. The C-index of external validations by itself showed 0.69 for OS (KROG 13-03), 0.65 for DMFS (KROG 12-08), and 0.85 for DFS (the SNU/AMC).

While the distribution of FIGO IIa in our Group A was similar to the KROG studies around 23%, many patients in Group B in our study had more advanced stages than in the SNU/AMC study, because the SNU/AMC study was focused not on adjuvant radiotherapy but on primary RH and PLND. The SNU/AMC study included 38% of FIGO stage Ia in comparison with 7% of our Group B. Nevertheless, the results were fair, and predictability of C-index was similar to ours. In addition, our study proved that the SNU/AMC nomogram was still effective for a cohort with adjuvant therapy after RH.

Our study showed poor prognosis in the young age group. It would be the most different point with the KROG 13-3 for OS. For age, various outcomes have been reported in other studies. In a Thai study of RH, an age of less than 35 years predicted poor survival [16]. In another surgical study, the increase of age decreased the DFS, with a hazard ratio of 0.935 with marginal significance (*P* = 0.052) [17], and, in a Japanese study using CCRT in huge FIGO IB–IIB cervical cancers, age less than 48 was an independent poor prognosis factor [18]. In contrast, in a pattern of care study in elderly patients, age over 70 years was a poor prognosis factor after adjusting for treatment disparities [19]. Two Western studies showed that the parameters of age over 65 years and 70 years were both poor prognostic factors [20, 21]. In nomogram studies, an Austrian study and a Taiwanese study presented contradictory outcomes for age [10]. We thought that perhaps age has a bimodal peak for poor prognosis, for both younger and elderly patients. One Korean nomogram suggested a similar bimodal finding for age. They gave a score of zero, intermediate, and high points for mid-age, <40 years, and >70 years, respectively [7]. Although further studies are needed, we expect that the score of age might be modified in the revised version of the nomogram.

Nodal metastases were important in our cohort. Our results showed that a group with lymph node metastases ≥3 showed the better 5-year survival than a group without lymph node metastases in OS, DMFS, and DFS. One study for RH and PLND reported that the more the number of lymph node metastases was, the poorer the survival was [22]. All of nomogram models after RH defined the number of pelvic lymph node metastases as a strong prognostic factor [12–14]. In addition, multiple groups of lymph node metastases were found to be a poor prognostic factor [23].

Although tumor size was masked in multivariate analyses of two KROG studies, tumor size is known as the determining factor of FIGO stage and it was an important prognostic factor in our cohort. While the histologic pattern in our study was insignificant, huge cohort studies in Taiwan and Korea reported that adenocarcinoma had poorer outcomes than squamous cell carcinoma [24, 25]. In addition, nonsquamous histology strongly influenced a nomogram model for DMFS with similar significance of two pelvic node metastases. In contrast, another study in Turkey reported that there are no difference between adenocarcinoma and squamous cell carcinoma [25]. For adenosquamous cell carcinoma, a Korean study suggested that the relapse-free survival is similar to that in squamous cell carcinoma [26]. In our study, the FIGO stage failed to achieve statistical significance. Most patients in our cohort were in FIGO stage Ib and the number of patients in other stages was relatively small; therefore, this finding probably would be associated with a distribution of cohort. The FIGO stage in two KROG studies did not have the significance in the same manner. Each prognostic factor such as parametrial invasion, lymphovascular invasion, and invasion thickness into uterine cervix was insignificant in our study. However, when these factors received the scores according to the intensity, we know that the overall predictability of prognosis can be improved and it was validated through C-index.

In addition to the nomogram models after RH in early uterine cervical cancer, a few other nomograms have been presented in definite radiotherapy. Two Korean studies evaluated the OS in groups treated with radiotherapy alone or with CCRT through a boot-strap method [7, 8]. A Taiwanese study evaluated the OS after CCRT through internal validation [9]. An Austrian study evaluated the OS in all patients after standard management, regardless of curative surgery or radiotherapy [10]. A recent study reviewed the patients enrolled in randomized Gynecologic Oncology Group studies and suggested nomograms for progression-free survival, OS, and pelvic recurrence [11]. The important factors for these studies were FIGO stage, tumor size, and lymph node status. All of the nomogram model developers claim that their models are better than FIGO stage for specific survival prediction after the standard therapeutic approach. These nomograms need to be additionally validated in various circumstances and be modified to improve the quality of the nomogram. In particular, since the endemic area of uterine cervical cancer is in developing countries, further validation should be done in these countries.

In conclusion, the published nomograms after hysterectomy were successfully validated in our study, with C-indices over 0.58. For personalized consultation with patients and a tailored therapeutic strategy, this information is available and is compatible with FIGO stage. For future clinical trials, these nomograms could be used as eligibility criteria. For example, the groups with low nomogram scores could be enrolled in studies to increase the intensity of adjuvant therapy, and vice versa. Also, some factors under debate in other studies could be continuously evaluated and modulated to make a more universal and suitable nomogram than the current version.

## Figures and Tables

**Table 1:** Patient's characteristics.

	Group A (*N* = 109)	Group B (*N* = 101)
Age		
Median (range) (years)	49 (28–73)	48 (28–73)

FIGO stage		
Ia : Ib: IIa	4 : 78 : 27	7 : 67 : 27

Histology		
Squamous : nonsquamous	86 : 23	101 : 0

Serum SCC antigen		
Missing	2	4
Median (Range) (ng/mL)	1.40 (0.1–29.2)	1.50 (0.1–29.2)

Tumor size		
≤4 cm : >4 cm	86 : 23	80 : 21

Depth of cervix invasion		
≤1/2 : >1/2	26 : 83	27 : 74

Parametrial invasion		
Negative : Positive	94 : 15	86 : 15

Resection margin involvement		
Negative : Positive	85 : 24	86 : 15

Lymphovascular invasion		
Negative : Positive	55 : 54	53 : 48

Pelvic lymph node involvement		
Negative : Positive	76 : 33	71 : 30

Concurrent chemotherapy		
No : Yes	29 : 80	31 : 70

External beam radiotherapy		
No : Yes	0 : 109	8 : 93
Median (range) (Gy)	50.4 (25.2–54)	50.4 (5.4–54)

Brachytherapy		
No : Yes	24 : 85	17 : 84
Median (range) (Gy)	21 (7–21)	21 (7–28)

**Table 2:** Log-rank tests for overall survival, distant metastases-free survival, and disease-free survival.

	5-year OS (%)	Log-Rank test *P* value	5-year DMFS (%)	Log-Rank test *P* value	5-year DFS (%)	Log-Rank test *P* value
Age (years)		0.029 (between all)		0.403 (between all)		0.200 (between all)
≤40	70.7	0.006 (≤40 : 41–64)	81.8	0.259 (≤40 : 41–64)	74.7	0.074 (≤40 : 41–64)
41–64	92.2	0.326 (≤40 : ≥65)	89.6	0.953 (≤40 : ≥65)	86.0	0.523 (≤40 : ≥65)
≥65	93.8	0.500 (41–64 : ≥65)	76.2	0.294 (41–64 : ≥65)	80.2	0.782 (41–64 : ≥65)

FIGO stage		0.481 (between all)		0.712 (between all)		0.335 (between all)
Ia	100.0	0.385 (Ia : Ib)	100.0	0.450 (Ia : Ib)	100.0	0.240 (Ia : Ib)
Ib	89.0	0.331 (Ia : IIa)	86.1	0.424 (Ia : IIa)	83.9	0.169 (Ia : IIa)
IIa	83.6	0.433 (Ib : IIa)	83.7	0.773 (Ib : IIa)	75.7	0.441 (Ib : IIa)

Histology		0.494		0.987		
Squamous	88.8		86.3			
Others	85.0		84.8			

Serum SCC antigen (ng/mL)		0.278		0.218		0.176
≤1.5	88.8		89.8		87.8	
>1.5	84.4		85.0		79.0	

Tumor size (cm)		<0.001		<0.001		0.058
≤4	94.7		92.5		86.7	
>4	64.3		59.6		66.7	

Depth of cervix invasion		0.162		0.300		0.255
≤1/2	100.0		90.3		87.6	
>1/2	84.1		85.1		81.5	

Parametrial invasion		0.093		0.383		0.300
Negative	90.7		87.1		84.6	
Positive	72.2		79.4		73.3	

Resection margin involvement		0.432		0.413		0.612
Negative	88.3		87.3		83.4	
Positive	86.7		79.4		80.8	

Lymphovascular invasion		0.417		0.080		0.124
Negative	92.4		90.8		86.6	
Positive	83.6		81.1		78.7	

Lymph node involvement		0.061 (between all)		0.010 (between all)		0.033 (between all)
0	92.8	0.265 (0 : 1-2)	89.6	0.551 (0 : 1-2)	87.8	0.604 (0 : 1-2)
1-2	82.6	0.009 (0 : ≥3)	87.0	0.001 (0 : ≥3)	83.3	0.006 (0 : ≥3)
≥3	60.0	0.407 (1-2 : ≥3)	55.6	0.098 (1-2 : ≥3)	50.0	0.174 (1-2 : ≥3)

Concurrent chemotherapy		0.895		0.554		0.871
No	92.8		82.0		82.5	
Yes	86.3		88.4		84.0	

OS: overall survival; DMFS: distant metastases-free survival; DFS: disease-free survival.

**Table 3:** Cox-regression analyses for overall survival, distant metastases-free survival, and disease-free survival.

	OS Hazard ratio (95% CI, *P* value)	DMFS Hazard ratio (95% CI, *P* value)	DFS Hazard ratio (95% CI, *P* value)
Age (years)			
≤40	4.63 (0.83–24.97, 0.097)		3.44 (0.99–11.91, 0.051)
41–64	Reference		Reference
≥65	1.38 (0.22–8.86, 0.734)		1.57 (0.32–7.82, 0.582)

Tumor size (cm)			
≤4	Reference	Reference	
>4	8.62 (2.73–27.03, <0.001)	5.13 (1.72–15.15, 0.003)	

Lymph node involvement			
0		Reference	Reference
1–2		3.48 (0.77–15.87, 0.105)	0.72 (0.17–2.98, 0.649)
≥3		4.03 (1.13–14.29, 0.031)	3.90 (1.08–14.07, 0.038)

OS: overall survival; DMFS: distant metastases-free survival; DFS: disease-free survival.

**Table 4:** The comparison of C-index between our hazard models and nomograms.

Survival		Our hazard model	Nomograms
Overall survival	C-index	0.671	0.612
	SE	0.044	0.036
	95% CI	0.585–0.758	0.541–0.683
	*P* value	<0.001	0.002
	*Difference of C-index*	*0.092*	
	*P value*	*0.071*	

Distant metastases-free survival	C-index	0.609	0.597
	SE	0.048	0.040
	95% CI	0.516–0.709	0.502–0.643
	*P* value	0.022	0.014
	*Difference of C-index*	*0.017*	
	*P value*	*0.897*	

Disease-free survival	C-index	0.565	0.587
	SE	0.034	0.037
	95% CI	0.499–0.631	0.514–0.661
	*P* value	0.055	0.020
	*Difference of C-index*	*−0.010*	
	*P value*	*0.842*	
